# Correction for Eddy Current-Induced Echo-Shifting Effect in Partial-Fourier Diffusion Tensor Imaging

**DOI:** 10.1155/2015/185026

**Published:** 2015-08-30

**Authors:** Trong-Kha Truong, Allen W. Song, Nan-kuei Chen

**Affiliations:** Brain Imaging and Analysis Center, Duke University Medical Center, 2424 Erwin Road No. 501, Durham, NC 27705, USA

## Abstract

In most diffusion tensor imaging (DTI) studies, images are acquired with either a partial-Fourier or a parallel partial-Fourier echo-planar imaging (EPI) sequence, in order to shorten the echo time and increase the signal-to-noise ratio (SNR). However, eddy currents induced by the diffusion-sensitizing gradients can often lead to a shift of the echo in *k*-space, resulting in three distinct types of artifacts in partial-Fourier DTI. Here, we present an improved DTI acquisition and reconstruction scheme, capable of generating high-quality and high-SNR DTI data without eddy current-induced artifacts. This new scheme consists of three components, respectively, addressing the three distinct types of artifacts. First, a *k*-space energy-anchored DTI sequence is designed to recover eddy current-induced signal loss (i.e., Type 1 artifact). Second, a multischeme partial-Fourier reconstruction is used to eliminate artificial signal elevation (i.e., Type 2 artifact) associated with the conventional partial-Fourier reconstruction. Third, a signal intensity correction is applied to remove artificial signal modulations due to eddy current-induced erroneous *T*_2_^*∗*^-weighting (i.e., Type 3 artifact). These systematic improvements will greatly increase the consistency and accuracy of DTI measurements, expanding the utility of DTI in translational applications where quantitative robustness is much needed.

## 1. Introduction

Diffusion tensor imaging (DTI) has proven a powerful tool for characterizing alterations of white matter microstructure [1–4]. DTI has been successfully applied to studies of human brain development [5–13] and various neurological and psychiatric diseases [14–16], including bipolar disorder [17, 18], Creutzfeldt-Jakob disease [19], gliomas [20, 21], depression [22], aphasia [23], Parkinson's disease [24], Alzheimer's disease [24, 25], and schizophrenia [23]. In order to quantify these changes (e.g., in terms of the fractional anisotropy or FA), voxel-wise signal variations across images obtained with different sets of diffusion-sensitizing gradients are fitted with a tensor model. It is also possible to fit the acquired data with alternative models that take non-Gaussian components into consideration, making it possible to resolve crossing fibers [26–29].

Since most of the MRI signal is attenuated by strong diffusion-sensitizing gradients, the signal-to-noise ratio (SNR) is inherently low in DTI data. This issue can be addressed with several approaches. First, the SNR can be improved by averaging multiple magnitude images from repeated DTI scans, at the expense of a prolonged scan time. Second, high-field systems (e.g., 7 Tesla) may be used to generate DTI data with an inherently higher SNR [30]. However, such systems are not available at the majority of clinical sites. Third, the SNR can be increased by shortening the echo time (TE), based on partial-Fourier echo-planar imaging (EPI), parallel imaging, or the combination of both (see Appendix). Another major benefit of using partial-Fourier EPI, instead of full-Fourier EPI, is that the signal decay within the acquisition window can be reduced and thus the point spread function (PSF) can be improved [31]. In fact, partial-Fourier EPI and parallel partial-Fourier EPI have become the most widely used protocols for DTI studies, because of their higher SNR as compared with full-Fourier EPI.

Even though partial-Fourier EPI and parallel partial-Fourier EPI can generate DTI data with a higher SNR, they are susceptible to various types of artifacts related to the* k*-space echo-shifting effect. It has been shown that the echo-shifting effect caused by intrascan head motion may result in artificial signal variations in DTI [32–34]. We have further used* k*-space energy spectrum analysis (KESA) to demonstrate that the echo-shifting-induced artificial signal loss (i.e., Type 1 artifact) in partial-Fourier EPI may not be corrected with postprocessing, while the echo-shifting-induced artificial signal elevation (i.e., Type 2 artifact) may be corrected with a multischeme reconstruction algorithm [35].

In addition to intrascan head motion, hardware-related factors (e.g., eddy currents induced by the diffusion-sensitizing gradients; concomitant fields) also produce a pronounced echo-shifting effect [36], which in turn generates three distinct types of artifacts in partial-Fourier DTI data. Even though the eddy currents may be globally reduced through calibrating the gradient preemphasis [37], this calibration may not effectively remove the spatially inhomogeneous eddy currents in different regions. Furthermore, the preemphasis calibration alone may not effectively address different sources of the echo-shifting effect in DTI data. Therefore, it is highly desirable to develop a new empirical approach to effectively remove the spatially inhomogeneous echo-shifting effects resulting from multiple sources. In this report, we will demonstrate that all three types of artifacts caused by the eddy current-induced echo-shifting effect can be effectively eliminated with a new DTI acquisition and reconstruction scheme. By using this improved and comprehensive methodology, high-quality and high-SNR DTI can be achieved reliably.

Since the eddy currents vary significantly among different MRI scanners, the presence of uncorrected eddy current-induced artifacts makes it difficult to achieve consistent diffusion measures in multicenter DTI studies. We thus expect that the improved acquisition and reconstruction methods proposed in this report will also improve the quantitative consistency of DTI measures for translational applications independent of imaging hardware and sites.

## 2. Theory and Methods

### 2.1. Quantification of the Echo-Shifting Effect Associated with Diffusion-Sensitizing Gradients in DTI

Even though most pulse sequences are designed such that the echo is expected to appear at the center of* k*-space, the echo may, in reality, deviate from the* k*-space center for different reasons. For example, susceptibility-induced magnetic field gradients may result in an echo displacement or echo-shifting effect in gradient-echo sequences [38–41], whereas intrascan head motion may result in an echo-shifting effect in spin-echo EPI-based DTI [34]. As shown in our previous paper, the echo-shifting effect along the phase-encoding direction resulting from intrascan head motion may result in Type 1 and Type 2 artifacts [35].

Here we will show that, in addition and similar to intrascan head motion, the eddy currents induced by strong diffusion-sensitizing gradients in DTI also produce a pronounced echo-shifting effect. For example, Figure 1(a) shows the central portions (16 × 16) of partial-Fourier DTI* k*-space data (with an acquisition matrix size of 96 × 76) obtained from a spherical gel phantom on a 3 Tesla MRI scanner (General Electric, Waukesha, WI, USA) with 15 diffusion-encoding directions, illustrating the impact of diffusion-sensitizing gradients on the echo-shifting effect along both readout (horizontal) and phase-encoding (vertical) directions. As demonstrated in the subsequent sections, the echo-shifting effect along the phase-encoding direction results in undesirable artifacts. Other scan parameters used in this single-refocused spin-echo DTI scan are listed in Section 2.5 (with only the first 10 slices shown in Figure 1).

Note that Figure 1(a) mainly shows the* k*-space echo distribution corresponding to the summed signals from all voxels in an individual slice but does not reveal the spatially dependent echo-shifting effect. In order to quantify the* k*-space energy displacement corresponding to different voxels, the KESA method should be employed (as described in [35, 40, 41]). This method can be summarized as a three-step procedure. First, *N*_*y*_ images are generated from the same *N*_*x*_ × *N*_*y*_ 2D* k*-space data by replacing different numbers *n* = 1, 2, 3, … *N*_*y*_ of *k*_*y*_ echo-lines with the values predicted from the remaining *N*_*y*_-*n *lines based on partial-Fourier reconstruction. Second, for a particular voxel, the signal variations across these *N*_*y*_ images are analyzed to identify the image that has the largest signal difference as compared with its neighboring images. As described in our previous papers, the echo-shifting effect associated with this particular voxel is equal to the number of truncated *k*_*y*_ lines in the identified image [40]. Third, a* k*-space energy displacement map, reflecting the echo-shifting effect for all voxels, can be obtained by repeating the second step for all voxels in the chosen slice.

Figure 1(b) shows the image-domain distribution of the* k*-space energy displacement along the phase-encoding direction (i.e., Δ*k*_*y*_ maps) obtained from the phantom DTI data. It can be seen that the echo-shifting effect is relatively uniform within each slice but varies significantly with the diffusion-encoding direction. In addition, for a particular diffusion-encoding direction (e.g., direction number 11), the echo-shifting effect varies more significantly among the odd slices (which were acquired first in this interleaved acquisition) than among the even slices, likely because the eddy currents have not yet reached a steady-state. Although the echo-shifting effect along the readout direction may also be quantified with the KESA method, it does not result in partial-Fourier DTI artifacts and thus is not quantified in this paper.

### 2.2. Correction for Type 1 Artifacts in Partial-Fourier DTI with* k*-Space Energy-Anchored Acquisition

In the absence of an eddy current-induced echo-shifting effect, the* k*-space energy peak (as schematically indicated by a red circle in Figure 2(a)) appears at the center of the targeted* k*-space coverage, where only a certain portion (gray area in Figure 2(a)) is acquired with partial-Fourier DTI. When there exists a pronounced echo-shifting effect due to eddy currents, the* k*-space energy peak may be shifted outside the acquisition window (Figure 2(b)), resulting in unrecoverable signal loss* post hoc*, which is termed Type 1 artifact. Here, we propose to address this issue with a* k*-space energy-anchored DTI acquisition approach. With a prior knowledge of the eddy current-induced echo-shifting effect, the prephasing gradient amplitude along the phase-encoding direction is modified such that the targeted* k*-space coverage moves with the* k*-space energy displacement (Figure 2(c)), thereby avoiding Type 1 artifacts. Note that different prephasing gradient values are used for different slices and different diffusion-encoding directions according to the corresponding echo-shifting effect. Since the eddy currents are subject-independent, this sequence may be optimized based on infrequently performed phantom calibration scans, where the echo-shifting effect corresponding to different slices and diffusion-encoding directions is quantified with the KESA method. Note that the infrequently performed phantom scan (e.g., once every 6 or 12 months) does not increase the clinical scan time and is thus practical for clinical utilization [42]. The eddy currents are spatially varying, so that the* k*-space energy peaks originating from different voxels within a slice may not always be refocused exactly at the center of the targeted* k*-space coverage (e.g., Figure 2(d)). Nevertheless, the variation of the echo-shifting effect corresponding to different voxels within an individual slice is insignificant (see Figure 1(b)), and Type 1 artifacts can be avoided as long as the* k*-space energy peaks remain within the acquired* k*-space.

### 2.3. Correction for Type 2 Artifacts in Partial-Fourier DTI with* k*-Space Energy-Anchored Acquisition and Multischeme Partial-Fourier Reconstruction

In partial-Fourier DTI, the intentionally omitted* k*-space data (yellow area in Figure 2(e)) can be estimated from the acquired data (light and dark gray areas in Figure 2(e)) by using partial-Fourier reconstruction, where the* k*-space data from the central band (dark gray area in Figure 2(e)) provide phase references. However, when the* k*-space energy peaks are shifted outside the central band due to eddy currents (Figure 2(f)), the conventional partial-Fourier reconstruction based on these phase references may result in an artificial signal elevation, termed Type 2 artifact. By using a* k*-space energy-anchored pulse sequence, Type 2 artifacts can be reduced or even potentially eliminated as schematically shown in Figure 2(g). However, since the eddy currents are spatially varying, the* k*-space energy peaks from certain brain regions may be located outside the central band even with a* k*-space energy-anchored acquisition, resulting in spatially variable Type 2 artifacts.

To address this issue, we propose to integrate the* k*-space energy-anchored acquisition and our previously developed multischeme partial-Fourier reconstruction [35], which can be summarized as a two-step procedure. First, the KESA method is used to identify the* k*-space energy peak locations corresponding to different voxels in the human brain DTI data obtained with the* k*-space energy-anchored pulse sequence. Second, for a particular voxel, the position and width of the central* k*-space band used for phase references in the partial-Fourier reconstruction are chosen based on the corresponding echo-shifting effect (measured from the KESA Δ*k*_*y*_ maps derived in the first step) to ensure that the echo peak is located at the center of the central band. Even though the effective spatial resolution along the phase-encoding direction varies spatially as a result of the echo-shifting effect inherent to DTI scans, images obtained from multischeme partial-Fourier reconstruction may be interpolated to the same spatial resolution. For example, the* k*-space data to be reconstructed with the scheme shown in Figure 2(h) can be zero-filled to the area enclosed by the green square.

### 2.4. Correction for Type 3 Artifacts in Partial-Fourier DTI with Signal Intensity Correction

It is well known that the echo-shifting effect in gradient-echo EPI results in variations of the effective TE and *T*_2_^*^-weighting, which in turn produce undesirable signal variations [39]. Similarly, the echo-shifting effect in spin-echo EPI also results in an additional and erroneous *T*_2_^*^-weighting, producing undesirable signal variations. Even though such signal variations may not be very significant in individual diffusion-weighted images, they actually result in severe artifacts when multiple images are combined to derive diffusion measures such as FA maps (as will be shown in the Results section). As schematically illustrated in Figure 3(a) and 1, the signal intensity (*S*_se_) of spin-echo EPI-based DTI data depends on the TE as a function of a *T*_2_ exponential decay (as indicated by a black dashed line in Figure 3(a)):

(1) $$\begin{array}{c} S_{\text{se}}=S_{0}\cdot \exp\left(-\frac{\text{TE}}{T_{2}}\right). \end{array}$$

In the presence of an eddy current-induced echo-shifting effect, the effective TE (TE_eff_) differs from the targeted spin-echo TE and the generated echoes have both *T*_2_- and *T*_2_′-weighting (i.e., the spin-echo becomes an asymmetric spin-echo), as schematically shown in Figure 3(b). The asymmetric spin-echo signal intensity (*S*_ase_) can be represented by either 2 or 3, where ΔTE = TE_eff_ − TE, depending on the direction of the echo displacement (see Figure 3(b)):

(2) Sase;R=S0·exp⁡−TE+ΔTET2·exp⁡−ΔTET2′;for  ΔTE>0

(3) Sase;L=S0·exp⁡−TE+ΔTET2·exp⁡ΔTET2′;for  ΔTE<0.

Equations 2 and 3 can be reformatted to 4 and 5, respectively:

(4) $$\begin{array}{c} S_{\text{ase};R}=S_{\text{se}}\cdot \exp\left(-\Delta \text{TE}\left(\frac{1}{T_{2}}+\frac{1}{T_{2}^{\prime }}\right)\right);\;\text{for}\;\;\Delta \text{TE}>0, \end{array}$$

(5) $$\begin{array}{c} S_{\text{ase};L}=S_{\text{se}}\cdot \exp\left(-\Delta \text{TE}\left(\frac{1}{T_{2}}-\frac{1}{T_{2}^{\prime }}\right)\right);\;\text{for}\;\;\Delta \text{TE}<0. \end{array}$$

The signal reduction (from *S*_se_ to *S*_ase_) resulting from the echo-shifting effect in DTI is termed Type 3 artifact and can be corrected by dividing the acquired signal intensity of each voxel by either exp⁡(−ΔTE/*T*_2_^*^) for ΔTE > 0 or exp⁡(−ΔTE/*T*_2_^−^) for ΔTE < 0, where 1/*T*_2_^*^ = 1/*T*_2_ + 1/*T*_2_′  and 1/*T*_2_^−^ = 1/*T*_2_ − 1/*T*_2_′ [43]. Note that ΔTE is spatially variable and is computed as the product of the EPI echo-spacing time and Δ*k*_*y*_, which is measured with the KESA method from the human DTI* k*-space data.

### 2.5. Phantom Experiments and Implementation of the* k*-Space Energy-Anchored DTI Pulse Sequence

DTI data were obtained from a spherical gel phantom on a 3 Tesla MRI scanner by using a single-refocused spin-echo partial-Fourier EPI sequence (i.e., with a Stejskal-Tanner diffusion preparation scheme) with the following scan parameters: TR = 4 s, TE = 103 ms, acquisition matrix size = 96 × 76 (i.e., partial-Fourier EPI with 28 overscans), voxel size = (2.5 mm)^3^, *b* = 1000 s/mm^2^, diffusion gradient amplitude = 40 mT/m, *δ* = 22 ms, Δ = 26 ms, inter-*k*_*y*_ echo-spacing time = 0.944 ms, 20 slices, and 15 diffusion-encoding directions. The acquired data were analyzed with the KESA method to quantify the echo-shifting effect corresponding to different slices and diffusion-encoding directions. Based on the derived KESA Δ*k*_*y*_ maps (Figure 1(b)), a* k*-space energy-anchored partial-Fourier EPI pulse sequence, with its phase-encoding prephasing gradient appropriately adjusted across slices and diffusion-encoding directions, was then implemented to minimize Type 1 artifacts.

### 2.6. Human Data Acquisition and Analysis

The proposed DTI acquisition and reconstruction methods were evaluated in three healthy volunteers on our 3 Tesla MRI scanner. Participants gave written informed consent for a protocol approved by the institutional review board of Duke University Medical Center. Six sets of DTI data were acquired from each participant by using both the conventional partial-Fourier EPI and the new* k*-space energy-anchored partial-Fourier EPI sequences, with three different acquisition matrix sizes: 96 × 64 (i.e., 16 overscans), 96 × 60 (i.e., 12 overscans), and 96 × 56 (i.e., 8 overscans) at TE = 82, 75, and 66 ms, respectively. Other scan parameters were identical to those used in the phantom scan.

The conventional partial-Fourier EPI data were reconstructed with Cuppen's algorithm [44]. The* k*-space energy-anchored EPI data (with Type 1 artifacts inherently eliminated) were reconstructed with the KESA-based multischeme partial-Fourier reconstruction method to correct for Type 2 artifacts, and the signal intensity correction was subsequently performed to minimize Type 3 artifacts. The signal intensity correction assumed average values of  *T*_2_ = 80 ms and *T*_2_^*^ = 47 ms for white matter at 3 T [45]. However, note that, even for a ±5 ms variation in *T*_2_ and *T*_2_^*^ values, the correction factors would only vary by about 3% for the largest eddy current-induced echo shift measured. Even though Cuppen's method was used in the current implementation of the multischeme reconstruction, other types of partial-Fourier reconstruction algorithm (e.g., homodyne reconstruction) can also be used in our multischeme reconstruction.

The consistency in the FA maps derived from the three conventional DTI data sets acquired with different matrix sizes was assessed by calculating the root mean square errors between all three possible pairs of FA maps (i.e., 16 versus 12 overscans; 12 versus 8 overscans; and 16 versus 8 overscans) and by subsequently averaging these three values. This procedure was then repeated for the three* k*-space energy-anchored DTI data sets acquired with different matrix sizes to assess the FA consistency in the improved DTI scheme.

We also acquired conventional partial-Fourier DTI data with a larger matrix size of 96 × 76 (i.e., 28 overscans) at TE = 103 ms. Four repeated scans were performed, so that the reconstructed magnitude data could be averaged to improve the SNR. Note that this data set, with a large number of overscans, has inherently low levels of Type 1 and Type 2 artifacts, even when processed with the conventional partial-Fourier reconstruction algorithm. Type 3 artifacts are also inherently lower in this data set acquired at a longer TE. Nevertheless, the very minor residual Type 2 and Type 3 artifacts were corrected with the multischeme partial-Fourier reconstruction and signal intensity correction, respectively, before averaging the four sets of magnitude images. The FA map generated from this averaged data set was then used as a reference to assess the accuracy of the other FA maps obtained at shorter TEs.

## 3. Results

Figure 4 compares diffusion-weighted EPI* k*-space data obtained from a representative subject using the conventional partial-Fourier acquisition scheme (top row) and the new* k-*space energy-anchored partial-Fourier acquisition scheme (bottom row) with different numbers of overscans (shown in three columns). This figure shows that, because of the eddy current-induced echo-shifting effect, the* k*-space energy peaks can easily be truncated in conventional partial-Fourier EPI, particularly with fewer overscans. Even though such an undesirable* k*-space energy truncation can potentially be avoided by using many overscans (e.g., full-Fourier or near full-Fourier EPI), the resultant SNR would be significantly reduced. On the other hand, by using the proposed* k*-space energy-anchored acquisition approach, the* k*-space energy peaks are consistently located at the center of the targeted* k-*space coverage, so that the* k*-space energy truncation can be minimized regardless of the number of overscans to avoid signal losses and ensure a high SNR.

Figure 5 compares diffusion-weighted images obtained with conventional partial-Fourier EPI (top row) and the improved acquisition and reconstruction methods (middle and bottom rows). Because of the echo-shifting effect in conventional partial-Fourier EPI, the images acquired with fewer overscans are significantly degraded by unrecoverable signal loss (i.e., Type 1 artifacts, as indicated by arrows in the top row). When reconstructing the* k*-space energy-anchored EPI data with the conventional Cuppen's algorithm, the resulting images are free from Type 1 artifacts but are still susceptible to artificial signal elevation (i.e., Type 2 artifacts) in several regions, as shown in the orange boxes in the middle row. Both Type 1 and Type 2 artifacts can be effectively eliminated when the* k*-space energy-anchored EPI data are reconstructed with the recently developed multischeme partial-Fourier reconstruction algorithm, as shown in the bottom row.

Figure 6 further illustrates the image quality improvement obtained after correcting for Type 3 artifacts, in addition to Type 1 and Type 2 artifacts. The top row of Figure 6 shows FA maps derived from conventional partial-Fourier EPI data acquired with three different numbers of overscans. As expected, the FA map with the fewest overscans has the most pronounced artifacts. As shown in the middle row of Figure 6, the FA maps are much more consistent when Type 1 and Type 2 artifacts are removed with the* k*-space energy-anchored acquisition and multischeme partial-Fourier reconstruction, respectively. However, there still exist errors in several areas (e.g., the red color indicated by arrows) due to signal intensity variations across different diffusion-encoding directions (i.e., Type 3 artifacts). After correcting for these residual artifacts, more consistent and accurate FA maps can be obtained from* k*-space energy-anchored EPI data for all numbers of overscans, based on the following assessments.

First, the averaged values of root mean squares, reflecting the FA inconsistency across the data obtained with different numbers of overscans, are 0.31 for conventional DTI (i.e., the top row of Figure 6), 0.13 for* k*-space energy-anchored DTI with Type 1 and Type 2 artifacts removed (i.e., the middle row of Figure 6), and 0.13 for* k*-space energy-anchored DTI with all three types of artifacts removed (i.e., the bottom row of Figure 6). In other words, as compared with the conventional partial-Fourier DTI, the level of FA consistency is improved by 58% after artifact correction using the developed methods.

Second, the FA inaccuracy in the nine FA maps shown in Figures 6(a) to 6(i), estimated by their root mean square errors relative to the low-artifact reference scan (i.e., with 28 overscans and 4 averages), is (a) 0.19, (b) 0.24, (c) 0.43, (d) 0.19, (e) 0.22, (f) 0.25, (g) 0.18, (h) 0.21, and (i) 0.24. In other words, as compared with the conventional partial-Fourier DTI, the accuracy level can be improved by 5.2% (for 16 overscans) to 44.2% (for 8 overscans) by using the improved acquisition and reconstruction scheme.

Third, it can be seen that the FA map obtained at short TE with the proposed DTI scheme (Figure 6(i)) has a higher SNR as compared with the FA maps obtained at long TE (Figures 6(a) and 6(g)), which appear to be more pixelated. Altogether, these results demonstrate that the artifacts caused by the eddy current-induced echo-shifting effect in partial-Fourier DTI can be effectively reduced with the new acquisition and reconstruction scheme, making it possible to simultaneously achieve high-quality and high-SNR DTI scans based on partial-Fourier EPI.

## 4. Discussion

The eddy currents and resulting DTI artifacts not only change with imaging parameters (e.g., TR, TE, diffusion-sensitizing gradient settings, number of overscans in partial-Fourier DTI), but also vary significantly from scanner to scanner, even when using identical scan parameters. As a result, eddy current-induced artifacts always contribute to the variations in diffusion measures across multiple scanners, making it difficult to design a standardized diagnostic DTI protocol that is consistent across multiple centers if only conventional acquisition and reconstruction methods are used. Here, we report an improved DTI acquisition and reconstruction scheme to minimize three distinct types of eddy current-induced artifacts existing in conventional partial-Fourier DTI. As demonstrated in this paper, when using the proposed DTI scheme, more consistent and accurate diffusion measures can be obtained from DTI scans acquired with different levels of eddy currents. We believe that the developed methods should prove highly valuable for generating reliable and consistent DTI data for translational applications and in multicenter trials.

Both Type 1 and Type 2 artifacts can inherently be minimized in full-Fourier DTI and conventional partial-Fourier DTI with a large number of overscans, however at the expense of an unavoidably longer TE and thus lower SNR (see Appendix). On the other hand, conventional partial-Fourier DTI data obtained with a smaller number of overscans have a higher SNR but are more susceptible to eddy current-induced artifacts. By using the DTI acquisition and reconstruction scheme presented in this paper, it is feasible to produce high-quality and high-SNR data with minimal eddy current-induced artifacts. Note that the goal of this paper is not to identify a universal set of DTI parameters (e.g., number of overscans) that optimizes both SNR and image quality, as the optimal parameters may vary for different DTI applications. The purpose of this paper is to demonstrate that it is feasible to minimize eddy current-induced artifacts in high-SNR partial-Fourier EPI with an improved acquisition and reconstruction scheme, rather than choosing a conventional full-Fourier or near full-Fourier DTI protocol that produces data with a lower SNR.

The* k*-space energy-anchored DTI pulse sequence, designed to eliminate Type 1 artifacts, relies on calibration data measured from a phantom scan. Since the eddy currents are relatively reproducible, these phantom calibration scans can be performed very infrequently (e.g., once every 6 or 12 months). Type 2 and Type 3 artifacts, on the other hand, are removed in postprocessing based on the information derived with the KESA method from the human brain DTI data. In addition to Type 1, Type 2, and Type 3 artifacts, eddy currents also result in geometric distortions and blurring artifacts, which we did not address in this work (e.g., the edge artifacts in the FA maps shown in Figure 6). However, additional distortion correction procedures may be incorporated to further remove such artifacts [46].

In this initial implementation, we used a phantom calibration with the same parameters (slice orientation, b-factor, diffusion-encoding directions, etc.) as the DTI scans to measure the eddy current-induced echo shifts. However, to avoid the need for a protocol-specific calibration and to increase the practical utility of the proposed method, future implementations may potentially use a single calibration to model the eddy currents and to compute the resulting echo shifts for different DTI protocols, as recently proposed for the correction of eddy current-induced distortions [47, 48].

It has been shown that the eddy currents can be reduced with a twice-refocused spin-echo DTI sequence [49, 50]. In this sequence, the diffusion-sensitizing gradient waveforms are designed to minimize eddy currents with intermediate decay rates, which contribute the most to temporal phase variations within the acquisition window and thus to geometric distortions in EPI [49]. However, eddy currents with short and long decay rates, which contribute significantly to the phase error accumulated prior to the EPI acquisition window and thus to the echo-shifting effect, may not always be nulled in twice-refocused spin-echo EPI. In this case, the improved DTI methods presented in this paper can also be applied to effectively remove the echo-shifting-induced artifacts in twice-refocused spin-echo DTI.

Finally, in this report, we only demonstrate artifact removal in partial-Fourier EPI data as a proof of concept. The proposed* k*-space energy-anchored acquisition and improved reconstruction procedures can be readily extended to parallel partial-Fourier EPI, where eddy currents produce the same three distinct types of artifacts.

## 5. Conclusions

In conclusion, we have developed an improved DTI acquisition and reconstruction methodology to effectively remove three distinct types of eddy current-induced artifacts commonly observed in conventional partial-Fourier DTI. We have also demonstrated that this new methodology can simultaneously achieve high-SNR and high-quality partial-Fourier DTI with a small number of overscans. This method is expected to greatly improve the quantitative consistency of DTI measures across subjects and sites, thereby extending the utility of DTI in translational applications at large.

## Figures and Tables

**Figure 1:**
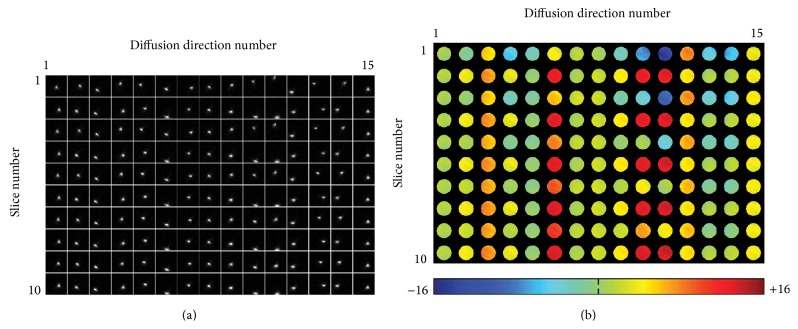
(a) The central portion of partial-Fourier DTI* k*-space data shows that the eddy current-induced echo-shifting effect varies with the diffusion-encoding direction and slice number. (b) The corresponding echo displacement maps, reflecting the spatially dependent echo-shifting effect along the phase-encoding direction, can be derived from the* k*-space energy spectrum analysis.

**Figure 2:**
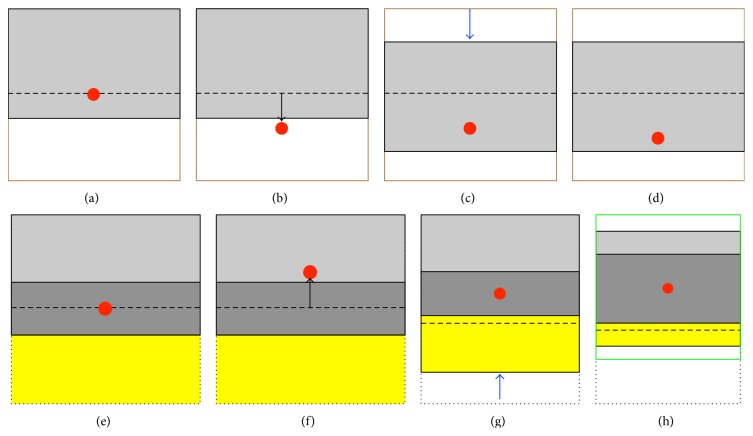
(a) The echo energy peak (indicated by the red dot) is expected to appear at the center of the targeted* k*-space coverage (enclosed by the solid black and brown lines) for partial-Fourier EPI (with the gray area representing the acquired data). (b) In the presence of an echo-shifting effect, the echo energy peak may be shifted outside the* k*-space acquisition area, resulting in signal loss (i.e., Type 1 artifact). (c) By using the proposed* k*-space energy-anchored pulse sequence, the* k*-space acquisition area (indicated by the gray rectangle) moves along with the eddy current-induced echo-shifting effect, avoiding Type 1 artifacts. (d) Even though the echo-shifting effect corresponding to different voxels within a slice may vary slightly, Type 1 artifacts can still be avoided as long as the echo energy peak remains within the* k*-space acquisition area. (e) The central *k*_*y*_ band (indicated by the dark gray rectangle) can provide phase references to estimate the signals in the omitted region (indicated by a yellow rectangle) in partial-Fourier reconstruction. (f) When the echo energy peak is shifted outside the central *k*_*y*_ band (while remaining within the* k*-space acquisition area), artificial signal elevation (i.e., Type 2 artifact) appears in the reconstructed images. (g) Type 2 artifacts may be reduced or even eliminated in data obtained with the* k*-space energy-anchored sequence. (h) Because of the spatially dependent echo-shifting effect, the echo energy peaks corresponding to different voxels within a slice may be shifted variably. In this case, the location of the central *k*_*y*_ band should be redefined (in postprocessing), according to the spatially dependent echo-shifting effect, to minimize Type 2 artifacts.

**Figure 3:**
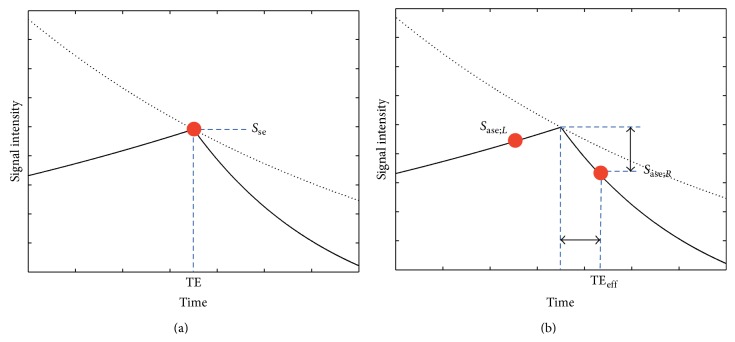
(a) The spin-echo DTI signal (indicated by the red dot) varies with the echo time according to a *T*_2_ decay curve (indicated by the dashed curve). (b) In the presence of an echo-shifting effect, the DTI signal is reduced by an additional *T*_2_′-weighting. *S*_se_ = spin-echo signal intensity 1, *S*_ase;*R*_ = asymmetric spin-echo signal intensity for ΔTE > 0  4, *S*_ase;*L*_ = asymmetric spin-echo signal intensity for ΔTE < 0  5. The horizontal arrow represents ΔTE and the vertical arrow represents the signal reduction from Type 3 artifacts (see text for details).

**Figure 4:**
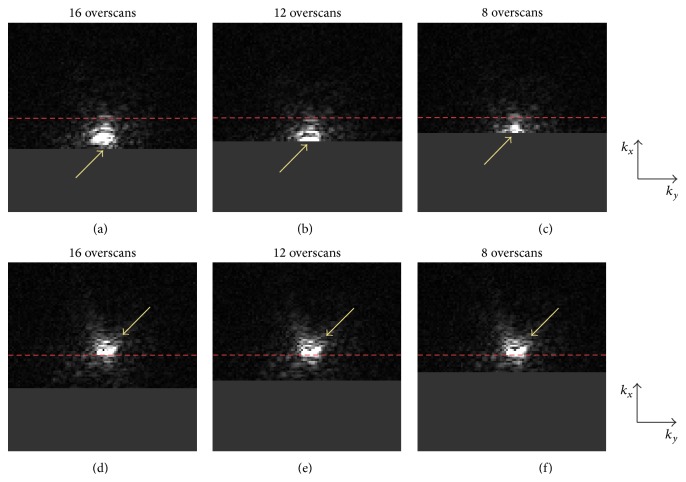
(a) to (c) show that the* k*-space data obtained with the conventional partial-Fourier DTI sequence, corresponding to different numbers of overscans, may be affected by a truncation artifact (i.e., Type 1 artifact) in the presence of an eddy current-induced echo-shifting effect. (d) to (f) show that this truncation artifact can be avoided in partial-Fourier DTI data obtained with the proposed* k*-space energy-anchored pulse sequence, where the* k*-space coverage moves with the echo-shifting effect.

**Figure 5:**
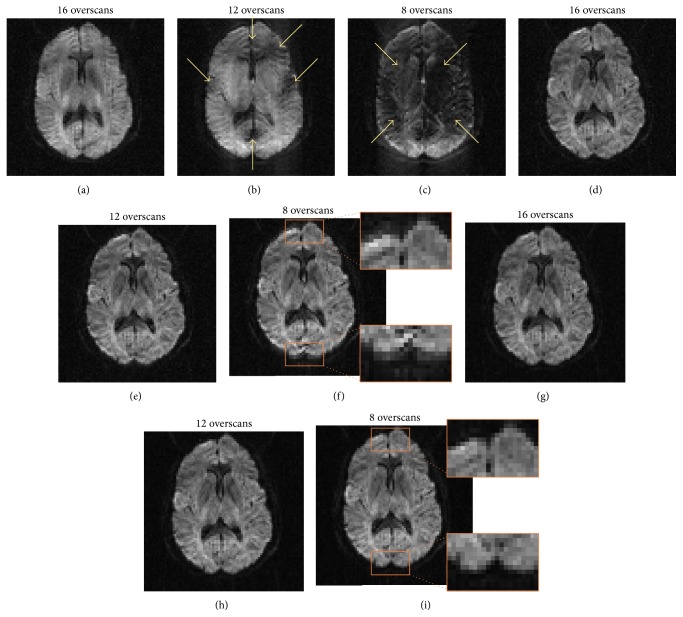
(a) to (c) show that the conventional partial-Fourier diffusion-weighted images are degraded by Type 1 artifacts, particularly for data obtained with a smaller number of overscans. (d) to (f) show that Type 1 artifacts can be avoided in data obtained with the* k*-space energy-anchored DTI sequence and reconstructed with the conventional partial-Fourier algorithm. However, artificial signal elevation (i.e., Type 2 artifact) appears in several regions. (g) to (i) show that both Type 1 and Type 2 artifacts can be eliminated when the multischeme partial-Fourier reconstruction algorithm is used to reconstruct the data acquired with the* k-*space energy-anchored pulse sequence.

**Figure 6:**
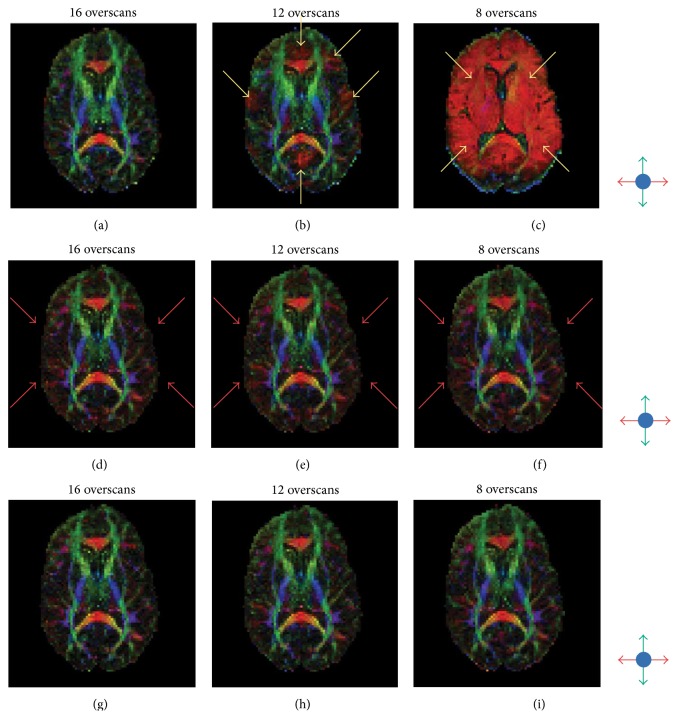
(a) to (c) show that the FA maps obtained with conventional partial-Fourier DTI and different numbers of overscans are highly inconsistent, due to the echo-shifting-induced Type 1 and Type 2 artifacts. (d) to (f) show that more consistent FA maps, across data acquired with different numbers of overscans, can be obtained after correcting for Type 1 and Type 2 artifacts. (g) to (i) show that the accuracy, quality, and consistency level can be further improved after correcting for Type 3 artifacts.

**Figure 7:**
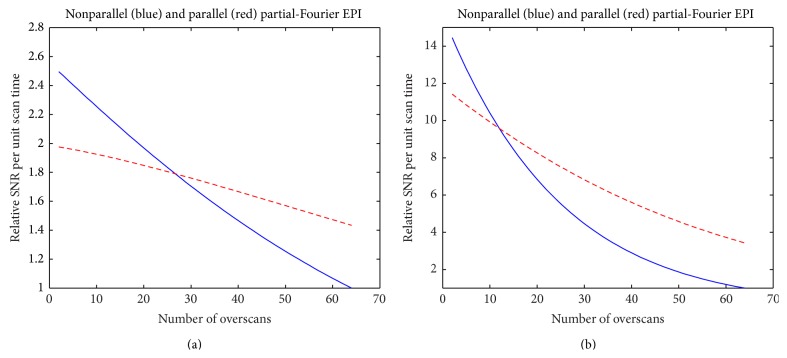
(a) The dependence of the relative SNR per unit scan time on the number of overscans for partial-Fourier EPI (blue curve) and parallel partial-Fourier EPI (red curve) of white-matter tissue (*T*_2_ = 80 ms). (b) The dependence of the relative SNR per unit scan time on the number of overscans for partial-Fourier EPI (blue curve) and parallel partial-Fourier EPI (red curve) of myelin (*T*_2_ = 30 ms).

## Appendix

We have performed a numerical analysis to compare the SNR per unit scan time corresponding to different acquisition schemes and to illustrate the need for using either partial-Fourier or parallel partial-Fourier EPI to improve the SNR per unit scan time, particularly for imaging tissues with short *T*_2_ components (e.g., myelin) [51]. Our analysis was performed based on actual imaging parameters obtained from our 3T GE system, using a well-known principle that the SNR per unit scan time in 2D imaging mainly depends on three factors: (1) the effective TE, (2) the number of phase-encoding steps, and (3) the minimally allowed TR. Basically, the SNR decays exponentially with the effective TE and is proportional to the square root of the number of acquired *k*_*y*_-lines (for a fixed voxel size). The SNR per unit scan time can then be subsequently estimated according to the minimal TR allowed, which is always much longer than the tissue *T*_1_ value for whole-brain DTI scans.

We have performed numerical simulations with different combinations of commonly used DTI scan parameters and have observed similar benefits of using partial-Fourier scans. For example, for whole-brain DTI scans with in-plane matrix size 128 × 128, FOV 24 × 24 cm,* b*-value 1000 s/mm^2^, and slice thickness 2 mm, the relative SNR per unit scan time achieved with partial-Fourier EPI with different numbers of overscans (in reference to full-Fourier DTI, i.e., to the data point with 64 overscans) is shown by the blue curve in Figure 7(a), where the white matter *T*_2_ value is assumed to be 80 ms. The SNR per unit scan time can be improved by more than 2-fold when using a small number of overscans (e.g., <10). The SNR per unit scan time achieved with parallel partial-Fourier DTI (with an acceleration factor of 2) corresponding to different numbers of overscans is shown by the red dashed curve in Figure 7(a) (also in reference to nonparallel full-Fourier DTI), illustrating that it is also beneficial to use a smaller number of overscans for parallel DTI.

Our numerical analysis further shows that the benefit of using DTI with a smaller number of overscans is even more pronounced for detecting the myelin signals (with *T*_2_ value < 35 ms). When assuming the tissue *T*_2_ value to be 30 ms, the SNRs per unit scan time for partial-Fourier DTI and parallel partial-Fourier DTI are shown by the blue and red curves in Figure 7(b), respectively. The SNR per unit scan time can be improved by more than 10-fold (in reference to full-Fourier DTI) when choosing a smaller number of overscans (e.g., <10) for imaging myelin.
